# System of models for simulation and optimization of operating modes of a delayed coking unit in a fuzzy environment

**DOI:** 10.1038/s41598-023-41455-0

**Published:** 2023-08-31

**Authors:** Batyr Orazbayev, Kulman Orazbayeva, Gulzhan Uskenbayeva, Elmira Dyussembina, Aliya Shukirova, Leila Rzayeva, Raigul Tuleuova

**Affiliations:** 1 Faculty of Information Technologies, L. N. Gumilyov Eurasian National University, Astana, 010000 Kazakhstan; 2 Faculty of Physics, Mathematics and Information Technology, Kh.Dosmukhamedov Atyrau University, Atyrau, 060011 Kazakhstan; 3 Faculty of Business, Esil University, Astana, 010000 Kazakhstan; 4 Department Intelligent Systems and Cybersecurity, Astana IT University, Astana, 010000 Kazakhstan

**Keywords:** Chemical engineering, Electrical and electronic engineering

## Abstract

The purpose of this study is to develop a method for synthesizing mathematical models of interconnected units of fuzzy chemical-technological systems (CTS) used for system modeling and optimization of their operating modes in a fuzzy environment. Since many CTSs in practice consist of many interconnected units, the development of their mathematical models combined into a single system of models, which allows systematic modeling and optimization of CTS parameters, is an urgent scientific and practical task. To develop a system of models of fuzzy described CTS, consisting of interconnected units, a system of methods is used that combines formal (experimental-statistical) and informal methods (methods of peer review, fuzzy set theory). A method for developing a system of mathematical models of CTS units under conditions of uncertainty due to the random and fuzzy nature of the available information is proposed. In the proposed method, mathematical models of various CTS units, depending on the nature of the initial and available information, are developed by various methods. Accordingly, various types of models are obtained, which are then combined into a single system of models, taking into account the interconnections of the system’s units. These results make it possible to develop more adequate models and determine the optimal CTS operating modes in a fuzzy environment by using the experience, knowledge and intuition of the decision maker, subject matter experts. Based on the proposed method, models of coke chambers and the main rectification column are developed in the form of combined models, including statistical and fuzzy models. The results obtained on the example of delayed coking units can be exported to similar CTS in oil refining, petrochemicals and other industries.

## Introduction

Technological processes of processing raw materials and manufacturing products in oil refining, chemical, petrochemical and other industries proceed in specially designed chemical-technological systems (CTS)^1,2^. Such CTS in practice consist of many interconnected process units with varying complexity, such as reactors, rectification columns, furnaces, heat exchangers, separators, and others. Depending on the complexity and availability of initial information about the state and modes of operation of such CTS units, their mathematical models can be developed based on various methods^3^. Moreover, many real CTS in practice operate under conditions of uncertainty associated with the random or fuzzy nature of the available information, which complicates the process of developing their mathematical models and optimizing their modes of operation. These CTSs include delayed coking units (DCUs) operating at the Atyrau refinery. DCU Atyrau refinery is designed for deep processing of oil refining residues (tar, fuel oil) and obtaining high-quality petroleum coke and other oil products from these oil refining residues, and operates under conditions of uncertainty^4,5^.

If the CTS uncertainty is caused by random values of the measured parameters, then the uncertainty problems can be solved based on the methods of probability theory and mathematical statistics^6–8^. If the uncertainties are caused by the fuzziness of the initial information, then to solve the problems of uncertainties, we need to apply the methods of expert assessments and the mathematical apparatus of fuzzy set theories^9–14^. In addition, fuzzy methods can be effectively used even in the presence of statistical, random data, since their collection and processing for complex CTS will often turn out to be very complex and not economically feasible. This situation substantiates the relevance of the task of developing a system of mathematical models of interconnected units of fuzzy CTSs, on the basis of which their operating parameters are optimized, and motivates the need for this study. Thus, the task of optimizing the DCU operating modes based on the system of models of its main technological units is currently an urgent scientific and technical problem.

Let us analyze the main research papers, which deal with the development of CTS mathematical models and optimization of their operating modes based on the developed models. Ibrahim D. and others investigated the issues of designing and optimizing the CTS of oil refining based on their models and a support vector machine^15^. The papers^16,17^ explored the problems of mathematical modeling and optimization of processes occurring in DCU and other CTS, taking into account random constraints, and methods for their solution based on an iterative approach. But in these and many other analyzed works^18–20^, the problems of developing models and optimizing CTS operating modes on their basis under conditions of uncertainty are not considered due to the fuzziness of some part of the initial information.

Li et al.^21^ investigated the problems of CTS column modeling based on the reaction kinetics and mass transfer process. Zhou et al., in the article^22^ investigated the methods of modeling and technical and economic analysis of the processes of obtaining liquid fuels at CTS based on an experimental-statistical approach, taking into account the uncertainty of a probabilistic nature. Methods for developing mathematical models of CTS technological units under conditions of uncertainty due to the fuzziness of the initial information and the problems of their multicriteria optimization and control are studied in monographs^23,24^ and in articles^25,26^. But in these and many other similar studies, the problems of developing a system of mathematical models of interconnected CTS technological units for system modeling of their work have not been investigated. In the studies of Zaichenko^27^, Liao et al.^28^ there were investigated the problems of fuzzy optimization and decision making in a fuzzy environment and proposed approaches to their solution based on the involvement of a decision maker (DM) and experts. In these and other works, for solving problems of fuzzy optimization and decision making, methods are proposed based on the transformation of the original fuzzy problem to a set of crisp problems using the α-level set. And then appropriate methods of precise optimization are applied to solve the resulting set of precise problems, the results obtained are then combined according to the rules of fuzzy set theory^27–30^. The main disadvantage of such methods is that most of the collected initial fuzzy information is lost, and this will lead to a decrease in the adequacy of the solutions obtained.

The problems discussed above in the analyzed works related to the development of a system of models of interconnected and technological CTS units and an adequate solution of optimization problems in a fuzzy environment motivate to conduct research aimed at solving them. In this regard, the purpose of this study is to develop a method for synthesizing a system of models of interconnected and mutually influencing technological units of complex, indistinctly described CTS for system modeling and optimization of their operating modes. Based on the proposed method, a system of models of the main interconnected DCU units is then developed based on the available experimental and statistical data and fuzzy information, which is the knowledge, experience and intuition of DM and experts. For computer simulation and optimization of DCU operating modes, the developed models are programmatically implemented using modern programming tools. The results obtained, implemented for DCU, can be exported to other CTS of oil refining, petrochemicals and other industries.

Equally, attempts to integrate models of interconnected CTS units, including DCUs, have not been undertaken. In this paper, we are talking about the application of the proposed method in section “A method for developing a system of models of interconnected technological CTS units based on various available information”, in order to develop a system of models of DCU units.

## Material and methods

The object of study of this work is the DCU Atyrau refinery, which refers to the complex CTS, which characterizes the fuzziness of some part of the initial information. To effectively optimize the DCU operating modes and the processes occurring in it, it is necessary to develop a system of mathematical models of the object of study. Such a system of models consists of combined models of the main DCU units and allows to systematically model and optimize its operating modes. In production conditions, to optimize delayed coking processes, it is necessary to develop and apply an optimization algorithm that, based on a system of models, allows choosing the optimal DCU operating modes^5^.

**Figure 1:**
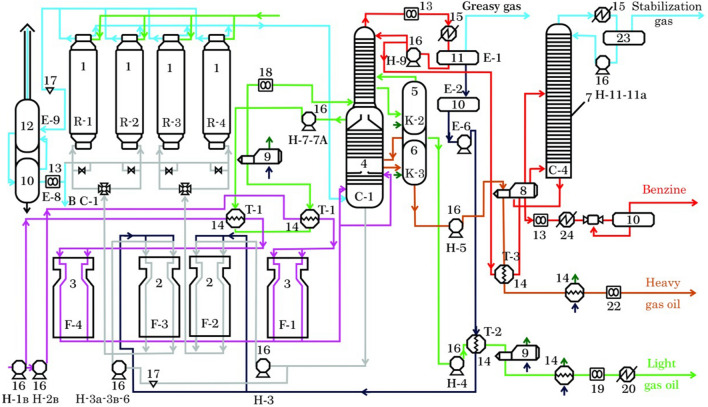
DCU 21-10/6 flow diagram. DCU 21-10/6 main units: 1—coking reactors (R-1 **–**R-4.), temperature in R-1, R-2, R-3 and R-4, 480−485 °С; pressure in R-1, R-2, R-3 and R-4, 4.1–4.8 kg/cm^2^; 2 and 3—furnaces for heating secondary and primary raw materials (F-2, F-3 and F-1, F-4), temperature in F-2, F-3, 385−398 °С, in F-1, F-4, 360–380 °С; pressure in F-2, F-3, 20−22 kg/cm^2^, in F-1, F-4, 7.1−7.5 kg/cm^2^; consumption of secondary raw materials in F-2, F-3, 20−22 t/h, in F-1, F-4, 28−30 t/h; 4—main rectification column (C-1), top temperature С-1, 118−120 °С, bottom temperature С-1, 365−370 °С; pressure in С-1, 4.0−4.5 kg/cm^2^; remote level С-1, 35–40 dm; 14—heat exchangers, temperature in heat exchangers, 120−140 °С.

The research materials in this paper are experimental and statistical data, theoretical information and expert information, presented mainly in the form of fuzzy information, describing the states and modes of operation of the DCU Atyrau refinery. This CTS is intended for deep processing of tar and production of petroleum coke from it, which is the target product, and other distillate products (gasoline, gas, light and heavy gas oils). Let us give a brief description of the delayed coking process according to the technological scheme of the object of study—DCU 21–10/6 Atyrau refinery, shown in Fig. 1.

Raw materials, i.e. the tar is heated in the raw material heating furnaces (F-1, F-4) to the required temperature and enters the main C-1 rectification column for separation into various fractions. In column C-1, heated primary raw material and vapors of oil products from coking reactors are separated into different fractions depending on the boiling point (gas, gasoline, light and heavy gas oils and secondary raw material). Gasoline, light and heavy gas oils from C-1 column are salable products, and the residues, i.e. secondary raw materials are heated to the required temperature in secondary raw materials furnaces F-2, F-3 and sent to coking reactors R-1–R-4. In these reactors, the process of delayed coking takes place and coke (target product) is produced with the required quality indicators, and also vapors of oil products are released, which are sent back for separation to C-1 column.

The quantity and quality of coke and other distillate products depend on the value of the input, operating parameters, i.e. on the operating modes of the DCU. This explains the often observed significant difference in the material balance of the delayed coking process^31–33^. Therefore, to select the optimal operating mode of the DCU, it is necessary to develop a system of models of its main units that describe the dependence of the quantity and quality of coke and other products on the value of the input and operating parameters of the DCU. Then, by implementing these models in the form of programs based on computer simulation of various modes of operation of the DCU, it is possible to determine the values of input, mode parameters that provide the optimal mode of operation of the object. The optimal mode of operation of the DCU is understood as one that allows obtaining the maximum volume of coke with specified quality indicators, taking into account the required volumes and qualities of other produced petroleum products.

The flow of information and the order of research in this work is carried out according to the following diagram, consisting of interconnected blocks:
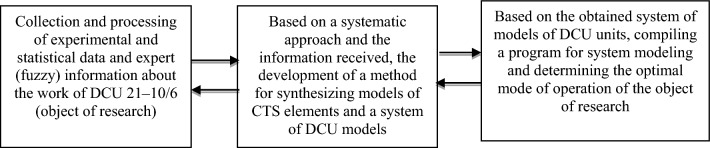


In more detail, the scheme of the research can be presented in the following sequence:

- the modes of operation of the DCU 21–10/6 Atyrau refinery were investigated, the collection and processing of experimental and statistical data on the operation of the object of study was carried out. At the same time, the following data on the operation and state of the DCU is collected, i.e. the values of the input, operating and output parameters of the main DCU units, characterized by the volume and quality of the DCU products:
  - for coking reactors R-1–R-4 ${x}_{1}$—volume of raw materials; ${x}_{2}, {x}_{3}$—temperature and pressure at the input of coking reactors; ${x}_{4}$—raw material coking index and ${x}_{5}$—recirculation rate, and ${y}_{1}$—the amount of coke produced, having numeric types;
  - for the main rectification column C-1 ${x}_{6}$—volume of raw material fed to C-1; ${x}_{7}$—the volume of vapors of petroleum products from coking reactor; ${x}_{8}$—volume of circulating irrigation; ${x}_{9}$—temperature of the upper part of C-1; ${x}_{10}$—gasoline output temperature having numeric types. Output parameters: ${y}_{5}, {y}_{6}, {y}_{7} \mathrm{and} {y}_{8}$—respectively, the volume of gasoline, light gas oil, heavy gas oil from the output of C-1 and residues from the bottom of the column, having numeric types;
  - for primary heating furnaces F-1, F-4 ${x}_{11}$—volume of raw material; ${x}_{12}$—temperature and ${x}_{13}$—pressure at their input, their output parameters: ${y}_{12}$—volume and ${y}_{13}$—temperature of heated raw material. For secondary raw material preheating furnaces F-2, F-3: ${x}_{14}$—volume of raw material; ${x}_{15}$—temperature and ${x}_{16}$—pressure at their input, their output parameters: ${y}_{14}$—volume and ${y}_{15}$—the temperature of the heated secondary raw material, having numeric types.
- by polling DM, experts, fuzzy information was collected, representing the experience, knowledge and intuition of DM, experts about the functioning of the DCU and processed by the mathematical apparatus of fuzzy set theories. At the same time, the fuzzy values of the following indicators of the main DCU units are evaluated:
  - for coking reactors R-1–R-4 ${\widetilde{y}}_{1}$—coke volume, ${\widetilde{y}}_{2}$—volatility and ${\widetilde{y}}_{3}$—ash content of coke having linguistic types;
  - for the main rectification column C-1 ${\widetilde{y}}_{9}$—gasoline quality from column C-1 (octane number), ${\widetilde{y}}_{10}, {\widetilde{y}}_{11}$—quality of light and heavy gas oil from output C-1 (boiling start) having linguistic types;
- on the basis of a systematic approach, a method is proposed for developing a system of models of interconnected CTS units based on available information of an experimental-statistical and fuzzy nature;
- based on the proposed method, a system of models of the main interconnected DCU units was developed;
- On the basis of the developed system of models of interconnected DCU units, software has been compiled that allows to systematically model the object of study on a computer and determine the optimal mode of its operation.

For the collection and processing of initial information, the development of mathematical models in a fuzzy environment and optimization, the methodology of system analysis^33,34^, experimental and statistical methods^6–8^, methods of optimization, decision-making^5,16,18,20,24–27^, methods of expert assessments and fuzzy set theories are used^10–14,23–25,27–30^.

Let's present the essence of the Delphi method used in expert assessment. The Delphi method is an iterative questionnaire procedure. At the same time, the requirement of the absence of personal contacts between experts and providing them with complete information on all the results of the assessments of each round of the survey is observed, while maintaining the anonymity of the assessments of argumentation and criticism. This eliminates conformity in expert assessment.

The essence of the Delphi method is a tool that allows to take into account the independent opinion of all members of a group of experts on the issue under discussion by consistently combining ideas, conclusions and proposals and come to an agreement. The method is based on multiple anonymous group interviews^9,10^.

The procedure of the method includes several successive stages of the survey. At the first stage, an individual survey of experts is carried out, usually in the form of questionnaires. Experts give answers without arguing them. Then the results of the survey are processed, the collective opinion of the group of experts is formed, the arguments in favor of various judgments are identified and summarized. At the second stage, all the information is given to the experts and they are asked to revise the estimates and explain the reasons for their disagreement with the collective judgment. New estimates are processed again and the transition to the next stage is carried out. As practice shows, after three or four stages, the experts' answers stabilize, and you can stop the procedure^11^.

The algorithm for organizing and conducting an expert assessment of the Delphi method consists of the following steps:

1. Form a working group to collect and summarize the opinions of experts.
2. Form an expert group of specialists who have questions on the topic under discussion.
3. Prepare a questionnaire, indicating in it the problem posed, clarifying questions. The wording should be clear and unambiguously interpreted, suggest unambiguous answers.
4. Conduct a survey of experts in accordance with the methodology, which, if necessary, repeats the procedure. The answers received serve as the basis for formulating questions for the next stage.
5. Summarize expert opinions and issue recommendations on the problem posed.

The advantage of the Delphi method is the use of feedback during the survey, which significantly increases the objectivity of expert assessments. However, this method requires considerable time to implement the entire multi-stage procedure. To reduce time, it is proposed to create and use a computer system.

### A method for developing a system of models of interconnected technological CTS units based on various available information

The proposed method for developing a system of models of interconnected CTS units is based on the methodology of system analysis and on the use of available information of a different nature. The developed method consists of the following main steps:

1. Study of CTS units, connections between them, collection and processing of available information, determination of the purpose of modelling.
2. Generation of criteria for evaluation and selection of models that can be developed for CTS units, taking into account the purpose of modeling. At the same time, in addition to standard criteria such as: availability of the necessary information; adequacy; the possibility of using for optimization, the cost of development and others, it is necessary to choose the possibility of combining into a single system of models as a criterion.
3. System analysis and expert evaluation according to the selected criteria for ranking models that can be developed for each unit of the system and, by the sum of the rank values, determine the optimal type of model for each CTS unit. This paragraph consists of the following subparagraphs.
4. If the theoretical information to describe the operation of the CTS unit is sufficient and, according to the sum of criteria values, i.e., according to the integrated criterion, the development of a deterministic model is effective, then a *deterministic model* is developed for this unit based on analytical methods. Then, to check the adequacy of the resulting model, go to step 11. Otherwise, go to the next step.
5. If statistical data to describe the operation of the CTS unit are available and the *statistical model* has the maximum value according to the integrated criterion, then the statistical models of this CTS unit are developed on the basis of experimental-statistical methods. Then, to check the adequacy of the resulting model, go to step 11. Otherwise, go to the next step.
6. If the theoretical and statistical data to describe the CTS unit are insufficient and the collection of such data is not economically feasible, then the possibility of synthesizing a fuzzy model is checked. If fuzzy information describing the operation of a given object under study is available, and according to the integrated criterion, the assessment of the fuzzy model will be maximum, then fuzzy models are developed for it based on the methods of fuzzy set theory. To do this, go to step 4;
7. If the theoretical, statistical and fuzzy information to describe the operation of the CTS unit is insufficient and the development of deterministic, statistical and fuzzy models for this object is impractical, then a *combined model* is developed for it by combining the available information of various types. In this case, various combinations of the initial available information (theoretical information, experimental and statistical data and fuzzy information) are used. To do this, depending on the nature of the initial information, go to paragraphs 3.1–3.3 or 4;
8. Determination of the fuzzy input, mode $\tilde{x}_{i} \in \tilde{A}_{i} ,\;i = \overline{{1,n}}$ and output parameters (criteria) $\tilde{y}_{j} \in \tilde{B}_{j} ,\;j = \overline{1,m,}$ necessary for the development of the model, where $\tilde{A}_{i} \in X,$ $\tilde{B}_{j} \in Y$—fuzzy subsets belonging to universal sets *X,Y*. Input, mode parameters can be crisp, i.e. $x_{i} \in X_{i} ,\;i = \overline{1,n}$. Here and below, ~ means the fuzziness of the corresponding sets and parameters. The input parameters for the models are selected taking into account their informativeness and influence on the operating modes, on the output parameters, which are the criteria for assessing the quality of the object's operation. And as the output parameters of the models, parameters are selected that determine the volume of products and quality indicators of the target product (coke, gasoline). When selecting the input and output parameters of the models, it is necessary to additionally take into account the expert information received from the DM and domain experts. In this process of input parameters for models, those parameters that have very little or no effect on the coking process can be neglected;
9. If the input, mode parameters of the CTS unit are crisp, i.e. $x_{i} \in X_{i} ,\;i = \overline{1,n,}$ then the structures of fuzzy equations of multiple regression are determined $\tilde{y}_{j} = f_{j} \left( {x_{1} , \ldots ,x_{n} , \tilde{a}_{0} ,\tilde{a}_{1} , \ldots ,\tilde{a}_{n} } \right),\;j = \overline{1,m} ,$ i. e. the problem of structural identification is solved. The structure of the fuzzy model can be identified based on the method of successive inclusion of regressors^34^. Otherwise, i.e. in the case of fuzziness and input, mode parameters $\tilde{x}_{i} \in \tilde{A}_{i} ,\;i = \overline{1,n}$ go to step 8.
10. Based on the methods of expert assessments with the involvement of DM, the object is described and the term-set of fuzzy parameters $T\left( {X_{i} ,Y_{j} } \right)$ is determined;
11. Construction of the membership function of fuzzy parameters $\mu_{{\tilde{A}_{i} }} \left( {\tilde{x}_{i} } \right)$, $\mu_{{\tilde{B}_{j} }} \left( {\tilde{y}_{j} } \right)$. To construct a membership function, for example, the output parameters of an object, it is proposed to use the following formula:
  1 $$\mu_{{B_{j} }}^{t} \left( {\tilde{y}_{j} } \right) = exp\left( {Q_{{\tilde{B}_{j} }}^{t} \left| {\left( {y_{j} - y_{j}^{md} } \right)^{{N_{{\tilde{B}_{j} }}^{t} }} } \right|} \right)$$

Here and below *t*—quantum number; $Q_{{\tilde{B}_{j} }}^{t}$—parameter characterizing the degree of fuzziness, its value is identified when constructing the membership function; $N_{{\tilde{B}_{j} }}^{t}$—a coefficient that allows to more accurately approximate the graph of the membership function; $y_{j}^{md}$—a fuzzy variable that more closely matches the selected term and is defined by the expression $\mu_{{\tilde{B}_{j} }} \left( {\tilde{y}_{j} } \right) = \mathop {\max }\limits_{j} \mu_{{\tilde{B}_{j} }}^{t} \left( {\widetilde{{y_{j} }}} \right).$ At this point, the membership function can be constructed by choosing a more appropriate function type from the typical functions of the Fuzzy Logic Toolbox of the MatLab graphic system, most often it is recommended to choose a Gaussian type function (**gaussmf**).

8. If the mode parameters and criteria, i.e., input $\tilde{x}_{i}$ and output $\tilde{y}_{j}$ parameters of the CTS unit, are described by linguistic variables, then fuzzy mappings are formalized $\tilde{R}_{ij} ,$ which determine the connections between $\tilde{x}_{i}$ and $\tilde{y}_{j}$*.* To construct linguistic models, go to step 10;
9. If the condition of paragraph 5 is satisfied, i.e., the input parameters are crisp $x_{i} \in X_{i} ,\;i = \overline{1,n,}$, then the values of fuzzy coefficients $\tilde{a}_{0} ,\tilde{a}_{1} , \ldots ,\tilde{a}_{n}$ of the models $\tilde{y}_{j} ,$ whose structures are identified in paragraph 5, are evaluated, i.e., the problem of parametric identification is solved, for example, based on the least square method^35^. Then go to step 11;
10. If the condition of paragraph 8 is satisfied, i.e. both the input and output parameters of the CTS unit under study are fuzzy, then based on the rules of compositional inference $\tilde{B}_{j} = \tilde{A}_{i}^\circ \tilde{R}_{ij}$ fuzzy values of the output parameters of the object are determined:
  2 $$\mu_{{B_{j} }}^{t} \left( {\tilde{y}_{j}^{*} } \right) = \mathop {\max }\limits_{{x_{i} \in X}} \left\{ {{\text{min}}\left[ {\mu_{{A_{i} }}^{t} \left( {\tilde{x}_{i}^{*} } \right),\mu_{{\tilde{R}_{ij} }}^{t} \left( {\tilde{x}_{j}^{*} ,\tilde{y}_{j} } \right)} \right]} \right\},$$
  where $$\mu_{{B_{j} }}^{t} \left( {\tilde{y}_{j}^{*} } \right)$$—membership function of fuzzy output parameters on the *t-*th quantum. Crisp, i.e., numerical values of the output parameters of the object $$\tilde{y}_{j}^{**}$$ are determined from the set of fuzzy solutions (2) by the formula:$$\tilde{y}_{j}^{**} = {\text{arg}}\mathop {\max }\limits_{{\tilde{y}_{j}^{*} }} \mu_{{B_{j} }}^{*} \left( {\tilde{y}_{j}^{*} } \right).$$
11. The adequacy condition of the model $R = \left( {y^{M} - y^{E} } \right)^{2} \le R_{D} ,\;$ is checked, where $R$ and $R_{D}$—the value of the criterion for assessing the adequacy and its acceptable value, $y^{M}$ and $y^{E}$—values of output parameters obtained from the model $\left( {y^{M} } \right)$ and from the results of experiments $\left( {y^{E} } \right)$. In this case, the values of $y^{M}$ and $y^{E}$ must be obtained with the same values of the input, mode parameters. If the adequacy condition is met, i.e. $R = \left( {y^{M} - y^{E} } \right)^{2} \le R_{D}$ then the developed models are considered adequate and are recommended for use. Otherwise, the cause of inadequacy is clarified and a return to the relevant items is carried out to eliminate the causes of inadequacy and achieve adequacy.

In the next section, based on the proposed and described method for developing a system of models of interconnected technological CTS units, based on the available statistical and fuzzy information from experts, we develop models of the main DCU units.

## Results

Let us present the results of the implementation of the main points of the method proposed above for developing a system of models of interconnected technological CTS units using the example of the synthesis of mathematical models of the main DCU units. The main interconnected technological units of the DCU, in which the main processes of delayed coking take place and oil products are obtained, are furnaces, the main rectification column and coking reactors. In paragraph 3 of the proposed method, as a result of system analysis and expert evaluation for the furnace, the main rectification column and the DCU coking reactors, effective types of models are determined by the values of the integrated criterion. At the same time, the concept of an integrated criterion means combining the value of all local criteria used to evaluate and select the best type of CTS units models into a single value, i.e. combining them into a common, one criterion. In our case, this is acceptable, since the values of local criteria used to evaluate the types of models are points from 1 to 5, which do not have a unit of measurement. Accordingly, the value of the integrated criterion is also points, meaning the sum of points of local criteria.

In the expert evaluation of the types of models for each unit, 10 experts participated, who evaluated according to a 5-score system. The results of system analysis and expert evaluation are given in the form of Table 1.

**Table 1:** Results of system analysis and expert evaluation of models of the main DCU units.

Main DCU units	Criteria for analysis and selection of the type of models	Types of models of the main DCU units			
		Deterministic	Statistical	Fuzzy	Combined
Coking rectors. R1– R-4, The developed models are given in section “Mathematical models of DCU coking reactors”	1. Availability of necessary information	2	2.5	4	4
	2. Possibility of use for optimization	3	4.5	4	4
	3. Adequacy of the developed model	4.5	3.5	4	3.5
	4. Model development cost	3	3.5	3	4
	5. Ability to combine into a single system of models	2.5	4	3.5	4.5
	Integrated criterion	15.0	19.0	18.5	**20.0**
Main rectification column, C-1 The developed models are given in section “Mathematical models of the main rectification column C-1 of the DCU”	1. Availability of necessary information	1.5	2	4	4,5
	2. Possibility of use for optimization	3	4.5	4	4.5
	3. Adequacy of the developed model	4.5	3.5	3.5	4.0
	4. Model development cost	4.5	4	4	3.5
	5. Ability to combine into a single system of models	4	4.5	3.5	3.5
	Integrated criterion	17.5	18.5	19.0	**20.0**
Heating furnaces, F-1,4; F-2,3 The developed models are given in section “Mathematical models of DCU heating furnaces”	1. Availability of necessary information	2.5	3.5	2.5	3.5
	2. Possibility of use for optimization	3	4.5	4.5	4
	3. Adequacy of the developed model	4	4	3	4.5
	4. Model development cost	3.5	4.5	3	3.5
	5. Ability to combine into a single system of models	4	4	3.5	3
	Integrated criterion	17.0	**20.5**	16.5	18.5

Note: The evaluation was carried out on a 5-point scale, where 1 is the lowest score, point; 5—the highest score, point.

Significant values are in [bold].

Next, we present the results of the development of selected types of models of the main technological units of DCU coking reactors R-1-R-4, of the main rectification column C-1 and primary (F-1.4) and secondary (F-2.3) heating furnaces (Table 1) according to the method proposed in section “Material and methods”.

As can be seen from Table 1, as a result of system analysis and expert assessment for coking reactors and the main rectification column, according to the value of the integrated criterion, the combined models were chosen as the most effective type of models for them. This is explained by the fact that in practice the necessary amount of relevant information is not enough to build deterministic, statistical or fuzzy models. Therefore, to construct these units, available information of a different nature is used (in our case, experimental-statistical and fuzzy). At the same time, combined models are understood as a system of models obtained on the basis of information of a different, for example, statistical and fuzzy nature.

Table 1 also shows that, according to the value of the integrated criterion for DCU preheating furnaces, statistical models were selected as effective ones. This is explained by the fact that for these objects, in practice, the necessary and reliable information of a statistical nature is available or can be collected experimentally.

### Mathematical models of DCU coking reactors

As a result of research and system analysis $${\mathbf{x}} = \left( {x_{1} ,x_{2} ,x_{3} ,x_{4} ,x_{5} } \right) _{ }$$ was defined as a vector of input, mode parameters that affect the coking process, the quantity and quality of coke, and the modes of operation of coking reactors. Components of the vector of input, mode parameters $${\mathbf{x}} = \left( {x_{1} ,x_{2} ,x_{3} ,x_{4} ,x_{5} } \right)$$ are: $$x_{1}$$—the volume of raw materials supplied to the coking reactors (heated raw materials from furnaces F-2.3, $$y_{13} ),$$ $$x_{1} =$$ $$y_{12}$$; $$x_{2} , x_{3}$$—temperature and pressure at the inlet of coking reactors; $$x_{4}$$—raw material coking index and $$x_{5}$$— recirculation ratio.

The target product of coking reactors is petroleum coke, the volume of which is indicated by $y_{1} .$ Qualitative indicators of coke, i.e. its volatility $\tilde{y}_{2}$ and ash content $\tilde{y}_{3}$ are characterized by fuzziness, since they are not directly measured, but are estimated indistinctly with the human mind. In addition, vapors of oil products—$y_{4} ,$ supplied from the coking reactors are fed to the main rectification column C-1 for the separation of distillate products.

In this study, for multidimensional modeling and optimization with fuzziness of some part of the initial information, the system method proposed by us above is used, which additionally uses fuzzy information based on peer review methods and fuzzy set theory. Structures of fuzzy models of coking reactors are identified on the basis of paragraph 5 of the method proposed above for developing mathematical models of CTS in the form of the following fuzzy multiple regression equations:

3 $$\tilde{y}_{j} = \tilde{a}_{0j} + \mathop \sum \limits_{i = 1}^{5} \tilde{a}_{ij} x_{ij} + \mathop \sum \limits_{i = 1}^{5} \mathop \sum \limits_{k = i}^{5} \tilde{a}_{ikj} x_{ij} x_{kj} , j = \overline{1,3, }$$

where $\tilde{y}_{j} , j = \overline{1,3}$—fuzzy output parameters: the volume of coke and its quality indicators, i.e. volatility and ash content; $x_{ij} ,x_{kj}$—input, mode parameters; $\tilde{a}_{0j} ,\tilde{a}_{ij} , \tilde{a}_{ikj}$—fuzzy regression coefficients that are subject to parametric identification.

The resulting fuzzy models (3) are then presented on the basis of the α-level set as a set of conventional (crisp) regression models that describe the effects of the vector ${\mathbf{x}} = \left( {x_{1} ,x_{2} ,x_{3} ,x_{4} ,x_{5} } \right)_{ }$ on the volume and quality of coke with different accuracy (depending on the α value):

4 $$y_{j}^{{\alpha_{q} }} = a_{0j}^{{\alpha_{q} }} + \mathop \sum \limits_{i = 1}^{5} a_{ij}^{{\alpha_{q} }} x_{ij} + \mathop \sum \limits_{i = 1}^{5} \mathop \sum \limits_{k = i}^{5} a_{ikj}^{{\alpha_{q} }} x_{ij} x_{kj} , j = \overline{1,3, }$$

In the system of Eqs. (4), the $\alpha_{q} :$ $L_{\alpha } = \left\{ {\alpha_{q} , q = \overline{1,3} } \right\}$ level set determines the reliability levels of the values of the regression coefficients of the model.

Then to identify fuzzy coefficients $\tilde{a}_{0j} ,\tilde{a}_{ij} , \tilde{a}_{ikj}$ of the models (3) it is necessary to determine the coefficients of crisp models (4) on the $\alpha :$ $a_{0j}^{{\alpha_{q} }} ,$ $a_{ij}^{{\alpha_{q} }} ,$ $a_{ikj}^{{\alpha_{q} }}$—level sets which satisfy the following condition at each α-level:

5 $$J_{j} = \mathop \sum \limits_{j = 1}^{3} \left( {y_{j}^{{\alpha_{q} }} - \hat{y}_{j}^{{\alpha_{q} }} } \right)^{2} \to {\text{min}}, q = \overline{1,3} ,$$

where $y_{j}^{{\alpha_{q} }}$—calculated values of output parameters obtained on the model, $\hat{y}_{j}^{{\alpha_{q} }}$—the real value of the output parameters obtained on the basis of expert information processing.

To take into account the more reliable part of the fuzzy information, the upper part of the membership function graph is selected, for example, three levels ($q = \overline{1,3}$): $\alpha = 0.5$, $\alpha = 0.8$ and $\alpha = 1$. Since the graphs of the membership function constructed according to the recommended formula (1) are bell-shaped and symmetrical, the following five values of the $\alpha$ level set are obtained: $\alpha = 0.5$, $\alpha = 0.8$ (left side of the graph of the membership function), $\alpha = 1$ and $\alpha = 0.5$, $\alpha = 0.8$ (right side of the graph).

The described approach allows solving the problem of identification of fuzzy coefficients of fuzzy model Eqs. (3) by reducing it to a set of crisp models (4) based on the α-level set. In this case, the identified fuzzy parameters $\tilde{a}_{0j} ,\tilde{a}_{ij} , \tilde{a}_{ikj}$ are determined on the basis of known methods of parametric identification at α-levels as crisp coefficients $a_{0j}^{{\alpha_{q} }} ,$ $a_{ij}^{{\alpha_{q} }} ,$ $a_{ikj}^{{\alpha_{q} }}$. Then, for computer simulation, the identified parameters at α-levels will be combined using the following expression:

6 $$\tilde{a}_{ij} = \mathop \cup \limits_{{\alpha \in \left[ {0.5 - 1} \right]}} a_{ij}^{{\alpha_{q} }} {\text{or}} \mu_{{\tilde{a}_{ij} }} \left( {a_{ij} } \right) = \mathop {\sup }\limits_{{\alpha \in \left[ {0.5 - 1} \right]}} {\text{min}}\left\{ {\alpha_{q} ,\mu_{{\tilde{a}_{ij} }}^{{\alpha_{q} }} \left( {a_{ij} } \right)} \right\},$$

where $a_{ij}^{{\alpha_{q} }} = \left\{ {\tilde{a}_{ij} |\mu_{{\tilde{a}_{ij} }} \left( {a_{ij} } \right) \ge \alpha } \right\}.$ Then the obtained crisp regression models, depending on the α value with different reliability, make it possible to estimate the volume of coke, its volatility and ash content at the outlet of the reactors.

In this work, the REGRESS software package, which implements the least square method^36^, was used to identify model parameters. This program interactively allows to identify the regression coefficients of nonlinear multiple regression equations with an arbitrary number of input parameters $x_{i} , i = \overline{1,n} .$

To identify clear coefficients of regression models using REGRESS programs, the collected statistical data on the operating mode of objects were processed and entered in batch mode into the $\alpha_{q}$ level program. In this program, the least square method is implemented, on the basis of which unknown regression coefficients are identified. To identify fuzzy regression coefficients, fuzzy models according to formula (4) are presented on level sets $\alpha_{q} , q = \overline{1,3} .$ Then, crisp values of fuzzy coefficients are identified on the $\alpha_{q}$ level using the REGRESS program and obtained a set of crisp values of fuzzy regression coefficients. For computer simulation, a set of crisp values of fuzzy regression coefficients are combined into a single value according to the formula (6).

After combining the obtained regression coefficients at α-levels according to formula (6), the following models are obtained, which are convenient for computer simulation and optimization of the operating modes of coking reactors.

1. Model estimating the volume of coke $\tilde{y}_{1}$ depending on the input, mode parameters $x_{1} ,x_{2} ,x_{3} ,x_{4} ,x_{5} :$
  7 $$\begin{aligned} \tilde{y}_{1} & = - 289.201 + 11.582x_{1} + 8.715x_{2} + 71.084x_{3} - 0.985x_{4} - 0.012x_{5} + 0.015x_{1}^{2} + 0.195x_{2}^{2} + 0.005x_{3}^{2} \\ & \;\;\; + 0.160x_{1} x_{2} + 1.500x_{1} x_{3} + 0.045x_{1} x_{5} + 1.223x_{2} x_{3} - 0.135x_{3} x_{4} - 0.033x_{3} x_{5} . \\ \end{aligned}$$
2. Model estimating the volatility of coke $\tilde{y}_{2}$ depending on $x_{1} ,x_{2} ,x_{3} ,x_{4} ,x_{5} :$
  8 $$\begin{aligned} \tilde{y}_{2} & = 59385 - 24.188x_{1} - 20.650x_{2} + 1.305x_{3} + 3.775x_{4} + 0.028x_{5} + 0.045x_{1}^{2} + 0.026x_{2}^{2} - 0.012x_{3}^{2} \\ & \;\;\; + 0.588x_{4}^{2} + 0.505x_{1} x_{2} + 0.045x_{1} x_{3} - 5.099x_{1} x_{4} - 0.197x_{2} x_{3} - 0.078x_{2} x_{4} . \\ \end{aligned}$$
3. Model estimating the ash content of coke $\tilde{y}_{3}$ depending on $x_{1} ,x_{2} ,x_{3} ,x_{4} ,x_{5} :$
  9 $$\begin{aligned} \tilde{y}_{3} & = 270.988 - 1.099x_{1} - 0.640x_{2} - 2.690x_{3} - 0.070x_{4} + 0.001x_{5} + 0.001x_{1}^{2} + 0.007x_{2}^{2} + 0.0001x_{3}^{2} \\ & \;\;\; + 0.025x_{4}^{2} - 0.062x_{1} x_{2} - 0.0006x_{2} x_{3} + 0.277x_{2} x_{4} + 0.029x_{3} x_{4} - 0.001x_{3} x_{5} . \\ \end{aligned}$$

The model for determining the volume of oil product vapors from the output of coking reactors $y_{4}$ was developed on the basis of experimental and statistical data and has the form of a regression model. After parametric identification of regression coefficients using the REGRESS software package we obtain:

10 $$\begin{aligned} y_{4} & = 157.165 + 8.717x_{1} + 3.445x_{2} - 28.085x_{3} - 12.070x_{5} + 0.998x_{1}^{2} + 0.557x_{2}^{2} - 0.156x_{3}^{2} + 0.334x_{1} x_{2} \\ & \,\;\; + 0.015x_{1} x_{3} + 0.185x_{1} x_{5} - 0.005x_{2} x_{3} + 0.0054x_{2} x_{4} + 0.004x_{3} x_{5} . \\ \end{aligned}$$

In the models developed above, obtained after parametric identification, the parameters of the regressors, which have little or no effect on the output parameters of coking reactors, are neglected.

### Mathematical models of the main rectification column C-1 of the DCU

When studying the main rectification column C-1 DCU, it was found that due to the complexity or impossibility of industrial measurement of such factors as the chemical and phase composition of raw materials, the levels of separation of products, the results of using traditional methods for modeling rectification columns are ineffective. These parameters, in the presence of experienced DMs, experts, can be fairly adequately estimated by fuzzy information. Therefore, in the conditions of fuzzy initial information for the development of models, we used the proposed method for developing models of technological objects based on available information of a different nature.

Let us consider the results of using the proposed method in the development of models of the main rectification column C-1 of the DCU. Based on system analysis and expert assessment, the following informative parameters have been selected that affect the separation process in C-1: $x_{6}$—the volume of raw materials supplied to C-1 (heated raw materials from furnaces F-1,4, $y_{12}$), i.e. $x_{6} = y_{12}$; $x_{7}$—the volume of vapors of petroleum products from coking reactors, $x_{7} = y_{4} ;$ $x_{8}$—circulating irrigation volume; $x_{9}$—temperature of the C-1 upper part; $x_{10}$—gasoline outlet temperature. Output parameters characterizing the quality of the rectification process: $y_{5} , y_{6} , y_{7} {\text{and}} y_{8}$—respectively, the volume of gasoline, light gas oil, heavy gas oil at the outlet of C-1 and residues from the bottom of the column. In addition, the output parameters of column C-1 are fuzzy estimated $\tilde{y}_{9}$—quality of gasoline from the column C-1, $\tilde{y}_{10} , \tilde{y}_{11}$—quality of light and heavy gas oil from the column C-1 (beginning of boiling). As a result of system analysis and expert evaluation of models of the main rectification column C-1, it was determined that the most effective for it is the development of combined models (Table 1), consisting of statistical and fuzzy models. Based on our proposed method for developing models of CTS units, based on the available information of a different nature, the structure and parameters of the rectification column C-1 of the DCU were identified.

The most effective models of the main rectification C-1 column are determined based on the criteria given in Table 1. Thus, the most effective models for C-1 are selected based on the following criteria: the availability of the necessary information for building the model; possibility of use for optimization; the adequacy of the developed model; the cost of developing a model and the possibility of combining the model being developed into a single system of models. The final choice of the model was made according to the maximum value of the integrated criterion, which was obtained by summing up the local criteria. At the same time, there were compromises between the adequacy and cost of the developed models, which were resolved in subsequent rounds of expert assessment using the Delphi method.

The structure of equations, which is a system of combined models (crisp models—to determine the volume of production and fuzzy models—to assess the quality of the product of the rectification column C-1), based on paragraphs 3.2 and 5 of the proposed method, is identified in the following form:

11 $$y_{j} = a_{0j} + \mathop \sum \limits_{i = 5}^{9} a_{ij} x_{ij} + \mathop \sum \limits_{i = 5}^{9} \mathop \sum \limits_{k = i}^{9} a_{ikj} x_{ij} x_{kj} , j = \overline{5,8, }$$

12 $$\tilde{y}_{j} = \tilde{a}_{0j} + \mathop \sum \limits_{i = 8}^{10} \tilde{a}_{ij} x_{ij} + \mathop \sum \limits_{i = 8}^{10} \mathop \sum \limits_{k = i}^{10} \tilde{a}_{ikj} x_{ij} x_{kj} , j = \overline{9,11} .$$

In the models (11, 12) obtained, all designations are described above.

For computer simulation, crisp and fuzzy regression coefficients were identified in accordance with paragraph 9 of the proposed method and using the REGRESS program. As a result of the identification of model parameters (10), we obtained:

13 $$\begin{aligned} y_{5} & = 11.805 + 0.625x_{6} + 0.842x_{7} - 0.434x_{8} - 0.561x_{9} + 0.195x_{10} + 0.003x_{6}^{2} + 0.002x_{7}^{2} - 0.0002x_{8}^{2} \\ & \;\;\; - 0.0001x_{9}^{2} + 0.00007x_{10}^{2} + 0.006x_{6} x_{7} - 0.001x_{6} x_{8} - 0.001x_{6} x_{9} + 0.002x_{6} x_{10} - 0.002x_{7} x_{8} - 0.001x_{7} x_{9} , \\ \end{aligned}$$

14 $$\begin{aligned} y_{6} & = 54.999 + 1.533x_{6} + 1.991x_{7} - 0.832x_{8} - 1.512x_{9} + 0.366x_{10} + 0.008x_{6}^{2} + 0.002x_{7}^{2} - 0.005x_{8}^{2} \\ & \;\; - 0.0006x_{9}^{2} + 0.001x_{10}^{2} + 0.002x_{6} x_{7} - 0.019x_{6} x_{8} - 0.007x_{6} x_{9} + 0.005x_{6} x_{10} - 0.013x_{7} x_{8} - 0.008x_{8} x_{9} , \\ \end{aligned}$$

15 $$\begin{aligned} y_{7} & = - 6.788 - 1.046x_{6} - 3.167x_{7} + 0.981x_{8} + 1.830x_{9} - 1.011x_{10} + 0.006x_{6}^{2} - 0.002x_{7}^{2} - 0.006x_{8}^{2} \\ & \;\;\; + 0.008x_{9}^{2} - 0.004x_{10}^{2} + 0.003x_{6} x_{7} - 0.002x_{6} x_{8} - 0.024x_{6} x_{9} + 0.005x_{7} x_{8} + 0.001x_{8} x_{9} - 0.009x_{9} x_{10} , \\ \end{aligned}$$

16 $$\begin{aligned} y_{8} & = 165.665 + 0.082x_{6} - 0.359x_{7} - 0.265x_{8} - 0.387x_{9} + 0.050x_{10} + 0.002x_{6}^{2} - 0.001x_{7}^{2} - 0.001x_{8}^{2} \\ & \;\;\; - 0.0003x_{9}^{2} + 0.004x_{10}^{2} - 0.007x_{6} x_{7} - 0.005x_{6} x_{8} - 0.002x_{6} x_{9} - 0.009x_{7} x_{8} + 0.001x_{7} x_{9} - 0.002x_{8} x_{9} , \\ \end{aligned}$$

After combining the values of fuzzy parameters of models (11) on the α-level set using formula (6), the following models, estimates of the quality of gasoline, light and heavy gas oils are obtained:

17 $$\begin{aligned} \tilde{y}_{9} & = - 32.399 + 1.926x_{8} + 0.610x_{9} - 0.192x_{10} - 0.293x_{8}^{2} + 0.262x_{9}^{2} \\ & \;\; + 0.003x_{10}^{2} + 0.003x_{8} x_{9} - 0.003x_{8} x_{10} + 0.001x_{9} x_{10} , \\ \end{aligned}$$

18 $$\begin{aligned} \tilde{y}_{10} & = 74.638 + 4.534x_{8} - 0.352x_{9} - 2.826x_{10} + 0.512x_{8}^{2} + 0.055x_{9}^{2} \\ & \;\;\; + 0.011x_{10}^{2} - 0.008x_{8} x_{9} - 0.005x_{8} x_{10} - 0.007x_{9} x_{10} , \\ \end{aligned}$$

19 $$\begin{aligned} \tilde{y}_{11} & = 54.303 - 0.922x_{8} - 0.318x_{9} + 0.786x_{10} + 1.909x_{8}^{2} + 0.128x_{9}^{2} \\ & \;\;\; + 0.011x_{10 }^{2} + 0.0005x_{8} x_{9} + 0.0002x_{8} x_{10} + + 0.003x_{9} x_{10} . \\ \end{aligned}$$

### Mathematical models of DCU heating furnaces

Since statistical data describing the operating modes of furnaces for heating primary F-1,4 and secondary raw materials F-2,3 are available, they can effectively develop statistical models (see Table 1). The input parameters that affect the process of heating and the operation of furnaces for heating primary raw materials are: $x_{11}$—volume of raw materials; $x_{12}$—temperature and $x_{13}$—pressure at their inlet, and the output parameters are: $y_{12}$—volume and $y_{13}$—temperature of the heated raw material. Similarly, the input parameters of furnaces for heating secondary raw materials: $x_{14}$—volume of raw materials; $x_{15}$—temperature and $x_{16}$—pressure at their inlet, and the output parameters are: $y_{14}$—volume and $y_{15}$—temperature of the heated secondary raw material.

On the basis of paragraph 3.2 of the proposed method and the experimental-statistical method, the structures of models of furnaces for heating primary and secondary raw materials are identified in the following form:

20 $$y_{j} = a_{0j} + \mathop \sum \limits_{i = 11}^{16} a_{ij} x_{ij} + \mathop \sum \limits_{i = 11}^{16} \mathop \sum \limits_{k = i}^{16} a_{ikj} x_{ij} x_{kj} , j = \overline{12,15, }$$

As a result of identifying the regression coefficients of furnace models (16) according to the processed statistical data and using the REGRESS program, we obtain

21 $$\begin{aligned} y_{12} & = 3.7785 + 0.5725x_{11} + 0.257x_{12} - 0.0885x_{13} + 0.025x_{11}^{2} + 0.091x_{12}^{2} \\ & \;\;\; + 0.001x_{13}^{2} + 0.0011x_{11} x_{12} - 0.0021x_{11} x_{13} - 0.0038x_{12} x_{13} , \\ \end{aligned}$$

22 $$\begin{aligned} y_{13} & = 18.001 - 0.0025x_{11} + 0.7547x_{12} - 0.303x_{13} - 0.0145x_{11}^{2} + 0.012x_{12}^{2} \\ & \;\;\; - 0.0078x_{13}^{2} + 0.003x_{11} x_{12} + 0.0125x_{11} x_{13} + 0.0017x_{12} x_{13} , \\ \end{aligned}$$

23 $$\begin{aligned} y_{14} & = 2.8857 + 0.6517x_{14} + 0.159x_{15} - 0.0778x_{16} + 0.033x_{14}^{2} + 0.001x_{15}^{2} \\ & \;\;\; + 0.002x_{16}^{2} + 0.0012x_{14} x_{15} - 0.0015x_{14} x_{16} - 0.0035x_{15} x_{16} , \\ \end{aligned}$$

24 $$\begin{aligned} y_{15} & = 17.003 - 0.003x_{14} + 0.689x_{15} - 0.255x_{16} - 0.0138x_{14}^{2} + 0.015x_{15}^{2} \\ & \;\;\; - 0.0067x_{16}^{2} + 0.0044x_{14} x_{15} + 0.0211x_{14} x_{16} + 0.0008x_{15} x_{16} , \\ \end{aligned}$$

### System of models of the main units of the installation for the optimal mode of operation of the DCU

**Figure 2:**
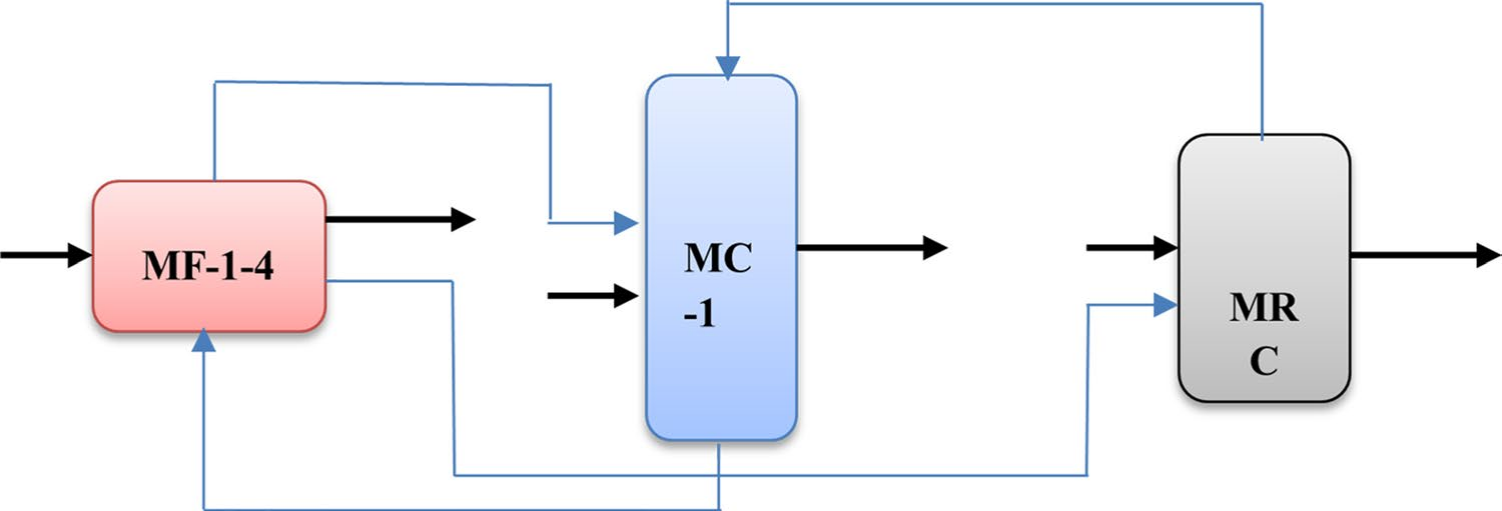
Scheme of combining models of the main DCU units into a single system of models.

For system modeling and optimization of DCU operating modes, it is necessary to create a package of models by combining the developed models of its main units. To this end, the developed models of coking reactors, the main rectification column and preheating furnaces are combined in accordance with their relationships and the course of the delayed coking process. The scheme for combining models of the main DCU units is shown in Fig. 2.

As can be seen from the diagram, the results of modeling one unit (model output) can be the input data for the model of another unit. For example, some results from furnace simulations (MF-1-4) is the input data for models of coking reactors (MRC), and part of the results of the MRC simulation is used as input data for models of the main rectification column. As a result of system modeling, it is possible to determine and select the optimal DCU operating modes according to the selected criteria. For automated search and determination of the optimal operating modes of the DCU, it is necessary to develop special decision-making algorithms that allow choosing the optimal operating mode of the object based on the considered models.

In the Fig. 2 MF 1–4, MC-1 and MRC—mathematical models implemented in the form of programs, respectively: preheating furnaces, main rectification column and coking reactors; —intermediate outputs of models passed as data to other models (simulation results); —initial data or final results.

### Computer simulation and optimization of DCU operating modes

To simulate and optimize the operating modes of the main units, a computer system was created in the RAD Studio 2010 environment, which is a product of Embarcadero Corporation, in the object-oriented Object Pascal language.

Let us present a computer simulation algorithm that was used to simulate the delayed coking technological process.

The proposed and used algorithm for computer simulation of the delayed coking process consists of the following main steps:

Step 1.: Based on a systematic approach and using the method proposed in section “A method for developing a system of models of interconnected technological CTS units based on various available information” for developing a system of models of interrelated technological CTS units based on various available information, develop models of the main DCU units (coking reactors, main distillation column and preheating furnaces).
Step 2.: On the basis of the scheme for combining the developed models into a single system of models, taking into account the course of the technological process shown in Fig. 2, combine the developed models into a single system of models. In this case, in accordance with the association scheme, the outputs of one model are used as input data for models of other objects.
Step 4.: For computer simulation, programmatically implement the resulting system of models in the form of a simulation system with a convenient user interface.
Step 5.: Based on the obtained simulation system and user interface, perform computer simulation of various DCU operating modes. At the same time, on a convenient user interface, the values of the input, mode parameters of the object change at their allowable change interval.
Step 6.: The user, changing the values of the input, operating mode parameters using the simulation system, explores various modes, i.e. analyzes the results obtained and determines when changing which parameters and in which direction the output parameters characterizing the quality of the object (criteria) improves.
Step 7.: Optimization of the DCU operating modes is carried out by computer simulation of various operating modes of the object, taking into account the imposed restrictions on the technological regulations of the object. When choosing the optimal operating mode of the object, the market demand for the manufactured products and the requirements for the quality indicators of the manufactured target products (petroleum coke, gasoline) are also taken into account.

Let us present the developed approach and the principle of its operation, revealing the details of the last step (step 7) of the computer simulation algorithm proposed above.

The essence of the approach to optimization is as follows. Using the created computer simulation system, the user, by changing the values of the input, operating parameters of the DCU units and observing the allowable intervals for their change, determines at what values of these parameters the output parameters are optimization criteria. As these criteria, the volumes and qualities of manufactured products that are in demand on the market (petroleum coke, gasoline) are used, determined using the model system developed in section “Results”.

The principle of optimizing the operating modes of the units and the delayed coking unit using the developed system of computer simulation and optimization is as follows. The user, who is a DM (DCU operator), maximizes the volume of the target products, i.e. petroleum coke and gasoline, taking into account their quality indicators. The main quality indicators that are taken into account during optimization are the volatility and ash content of coke, as well as the octane number of gasoline. In addition, when optimizing, DM must take into account the market demand for manufactured products and the requirements imposed by consumers on the quality indicators of manufactured target products (petroleum coke, gasoline).

Here is a description of the main interfaces of the created computer system based on the developed system of models of interconnected CTS units, which makes it possible to systematically simulate its operation and determine the optimal mode of operation of the DCU.

**Figure 3:**
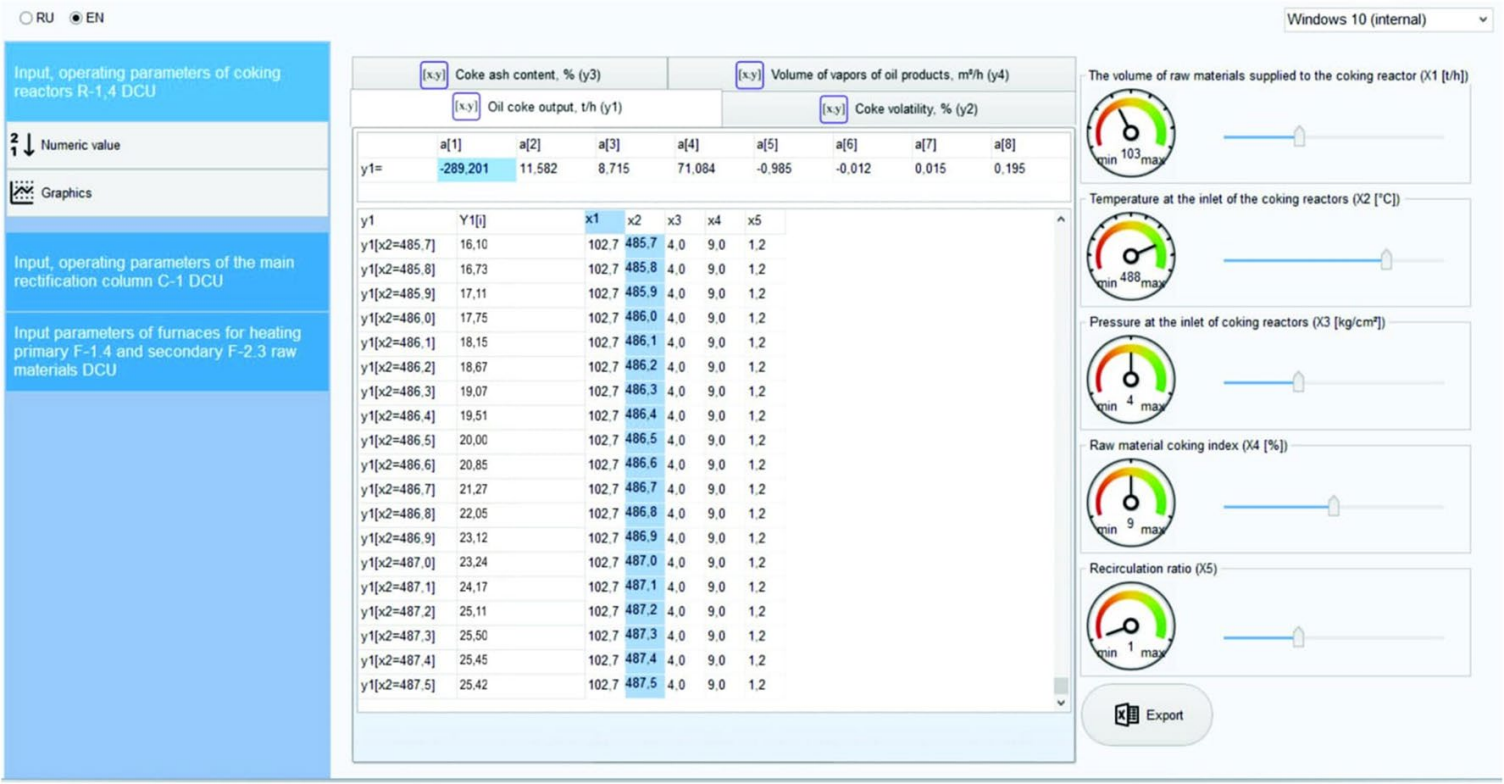
System interface for simulation and maximization of coke yield.

**Figure 4:**
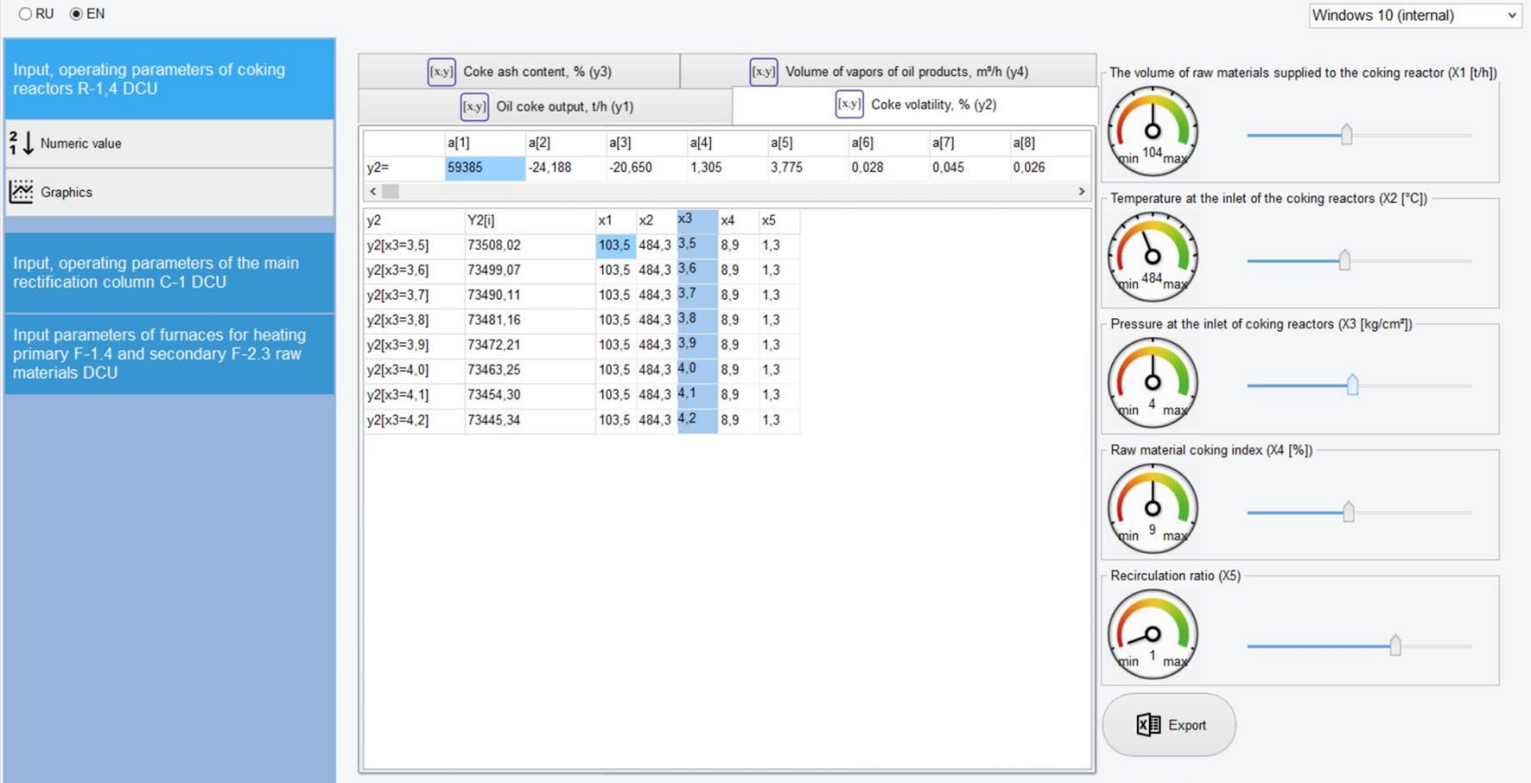
System interface for simulation and optimization of coke volatility.

The main window of the program, which is a graphical user interface, is shown in Fig. 3. As can be seen from the figure, the user interface is divided into 3 blocks. The first block contains menus for selecting the simulation object: R-1–R-4 DCU coking reactors; main rectification column C-1; furnaces for heating primary F-1,4 and secondary raw materials F-2,3. The second block contains the names of the output parameters of the selected simulation object, the identified parameters of the models, and the simulation results are displayed. The third block presents the input, regime parameters of the selected simulation object with the possibility of changing their values. Figures 3 and 4, as an example, show the results of selecting and modeling coking reactors to search for and determine their optimal operating modes, which ensure the optimization of the yield of the target product—coke (${\widetilde{y}}_{1}$) and its main quality indicator—volatility (${\widetilde{y}}_{2}$).

To check models using computer simulation, the model systems obtained in sections “Mathematical models of DCU coking reactors”–“System of models of the main units of the installation for the optimal mode of operation of the DCU” are programmatically implemented in the RAD Studio 2010 environment and a convenient user interface has been created. The user, using the user interface, by changing the values of the input parameters of the object, i.e., simulating various modes and analyzing the results obtained (values of the output parameters), selects the effective operating mode of the object. An analysis of the simulation results showed that the discrepancy between the calculated (predicted) and actual values of the output parameters is 3.4% (Table 2). This discrepancy is quite acceptable, since when modeling industrial facilities, the discrepancy between the calculated ones obtained on the models and the actual data is allowed up to 5%.

**Table 2:** Comparison of simulation results based on the developed system of models, known deterministic, statistical models and production and experimental data with DCU Atyrau refinery.

DCU output parameters (criteria)	Simulation results by known models from sources^33,37^	Simulation results based on the developed system of models in a fuzzy environment	Production (real) data obtained experimentally
Oil coke yield, $\tilde{y}_{1} ,$ t/h	22.00	25.50	24.85
Volatility of coke, $\tilde{y}_{2}$, %	–	8.70	(9.00)^L^
Ash content of coke, $\tilde{y}_{3}$, %	–	0.27	(0.28)^L^
Vapor volume of petroleum products, $y_{4} ,$ m^3^/h	5.35	5.43	5.45
Gasoline yield, $y_{5}$, t/h	9.80	10.75	10.30
Light gas oil yield, $y_{6} ,$ t/h	36.50	35.70	35.20
Heavy gas oil yield, $y_{7} ,$ t/h	32.80	27.20	30.50
Secondary raw materials yield, $y_{8} ,$ t/h	27,50	29,70	29.10
Gasoline quality, $\tilde{y}_{9} ,$ °C	−	51	(51.3)^L^
Light gas oil quality, $\tilde{y}_{10} ,$ °C	−	175	(175)^L^
Heavy gas oil quality, $\tilde{y}_{11} ,$ °C	−	230	(230)^L^
Heated primary raw material yield, $y_{12} ,$ t/h	103	103.05	103.50
Temperature of heated raw material, $y_{13} ,$ °C	390	385	386
Heated secondary raw material yield, $y_{14} ,$ t/h	27.10	29.70	29.60
Temperature of heated secondary raw material, $y_{15} ,$ ^o^C	495	493	493

Note: The values of the input, mode parameters of models and experiments are taken the same; (⋅)^L^ means that the values of these parameters are determined in laboratory conditions with human participatio; – means that the values of these parameters in this model are not determined.

The results of computer simulation and optimization of DCU operating modes based on the obtained system of models of its main units using available experimental-statistical and fuzzy information and modeling based on known models^33,37^ are shown in Table 2 for comparison. This table also contains real data obtained experimentally on the object with the same values of the input, mode parameters used in the simulation.

**Figure 5:**
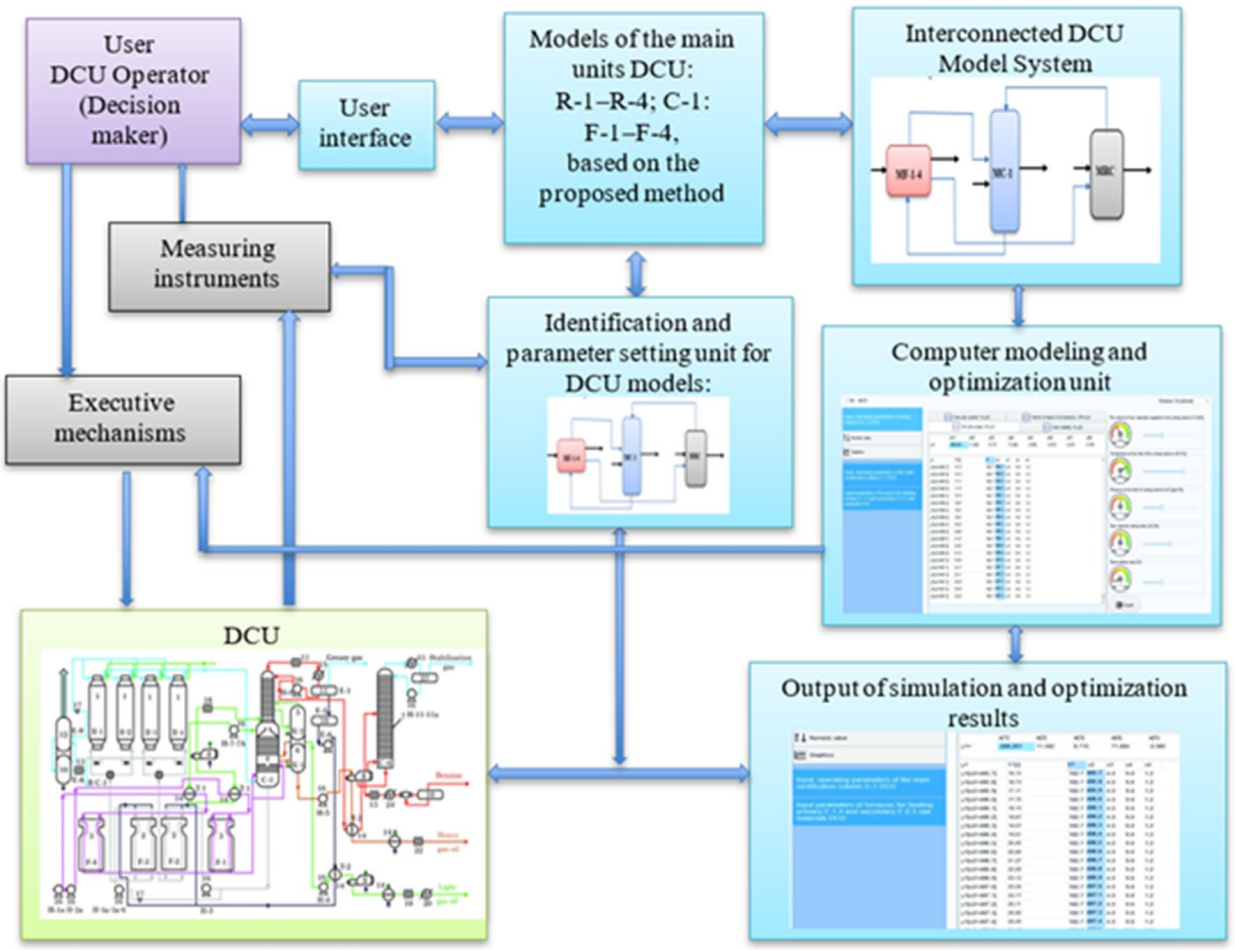
Computer simulation and optimization system for DCU operating modes and its interconnected components.

For a clearer explanation of the proposed approach to improving the current DCU operating mode control system through system modeling and optimization of the object operating modes, we present the following diagram (Fig. 5) and its description.

Let us consider the description of the proposed system for improving the modeling and optimization of DCU operating modes. In the (Fig. 5) diagram, the components of the existing system are shaded in gray, the common components in green, and the components of the proposed system in blue. There was no computer modeling and optimization unit in the existing control system of the DCU operating modes of the Atyrau Oil Refinery. In the existing system, operators-DM, which controls the operating modes of the object, receiving information about the state, values of the parameters of the object from the measuring instruments and, based on their experience, knowledge and intuition, made decisions on operating modes control. Naturally, less experienced operators managed the operating modes inefficiently, many problems arose. In this regard, it became necessary to develop a computer simulation and optimization system that allows DM to quickly make an effective decision on controlling the DCU operating modes based on simulation.

The proposed system for computer simulation and optimization of DCU operating modes is implemented as follows. The system user (DM), using models of the main DCU units integrated into the system of models, simulates various modes of operation on a computer and, analyzing the results obtained, selects the most efficient operating mode of the object. Then, to optimize the operating mode, he implements the decisions made through the executive mechanisms. To set up and identify the parameters of the unit models, when the state of the object and the composition of the raw material change, a unit for identifying and setting the parameters of the DCU models is introduced into the system. For the analysis and output of the obtained results, a unit for outputting the results of modeling and optimization is used. For convenient and effective interaction between users and the system, a friendly user interface is used.

## Discussion

The method proposed in section “Material and methods” for developing a system of models of interconnected technological CTS units is based on the methodology of system analysis and the use of available information of a different nature. The method, depending on the nature of the available initial information, allows developing deterministic, statistical, fuzzy, linguistic or combined (hybrid) models of individual DCU technological units. Evaluation of the effectiveness of the selected type of models for CTS units is based on system analysis and expert assessment (Delphi method) for a variety of criteria and multi-criteria selection. Then the developed models according to the proposed scheme are combined into a single system of models for computer simulation and optimization of the operating modes of the object of study. The choice of combined models as effective models for the main rectification column and DCU coking reactors is explained by the complexity of these units and the lack of deterministic and statistical information and/or the economic inexpediency of obtaining such information. In this regard, fuzzy information and other types of available information are used for these units to develop their models, which are used in combination based on appropriate methods (see paragraph 3.4 of the proposed method).

Since the yield and quality indicators of petroleum coke from coking reactors and the quality of products from the main rectification column (gasoline, light and heavy gas oils) are not directly measured, but are assessed with the participation of a person in laboratory conditions, they are characterized by fuzziness. Therefore, models estimating the volume, volatility and ash content of coke ($\tilde{y}_{1} \tilde{y}_{3}$), the quality of gasoline, light and heavy gas oils are developed in the form of fuzzy regression equations ($\tilde{y}_{9} \tilde{y}_{11}$). Mathematical models for determining the volumes of oil vapors from coking reactors, products from the output of the C-1 DCU column are identified in the form of statistical models and in the form of equations of polynomial type ($y_{4}$–$y_{8}$, $y_{12}$–$y_{15}$). Thus, for the coking reactors and the DCU main rectification column, combined models have been developed based on statistical and fuzzy information.

In the proposed method, due to the synergy effect and the emergence property of the system of methods and models used, it made it possible to solve the problems of scarcity and fuzziness of the initial information and obtain effective models of DCU units. The solution of the problem of optimization of the delayed coking process is carried out on the basis of the developed system of mathematical models by computer simulation of various modes of operation of the DCU and comparing the results. This approach is quite effectively implemented on the basis of modern computers with high performance.

As can be seen from the created scheme for combining models of the main DCU units into a single system of models (Fig. 2), the results of modeling one unit (model output) can be the input data for the model of another unit. For example, some results from furnace simulations (MF-1-4) are input data for coking reactor (MRC) models, and some of the MRC simulation results are used as input data for main rectification column models (MC-1). As a result of system modeling, it is possible to determine and select the optimal DCU operating modes according to the selected criteria. Research results obtained for DCU can be exported to similar CTS of oil refineries, petrochemicals and other industries.

As a result of the discussion of the obtained simulation results in order to determine the optimal DCU operating mode based on the known and developed system models using fuzzy information given in Table 2, the following advantages of the proposed system approach can be distinguished:

1. The proposed systematic approach to CTS modeling, taking into account fuzzy information, in comparison with known models, describes the real situation more adequately. At the same time, the results of the proposed method of system modeling in a fuzzy environment, in comparison with the results of known models, more accurately match the real production data obtained experimentally.
2. The proposed systematic approach to the development of models, modeling and optimization of CTS operating modes allows to determine more optimal modes of its operation. For example, based on computer simulation and optimization with the help of the developed models, the coke yield is increased by 0.65 t/hour or by 2.55% per hour. The yield of gasoline, which is also an important product, is also increased by 4.19% or more than the results of known methods. Accordingly, this allows to get a significant economic effect from the sale of target products.
3. A very important task of modeling and optimizing production facilities is to determine and control the quality of manufactured products. The developed method of computer simulation and optimization, taking into account the fuzziness of the initial information, makes it possible to evaluate the quality indicators of the produced target products (${\widetilde{y}}_{1}-{\widetilde{y}}_{3}, {y}_{12}$−${y}_{15}$), which are not determined by known methods. At the same time, the quality indicators of the target CTS products are evaluated based on the experience, knowledge and intuition of DM, experts, which is an important advantage of the proposed approach.

When modeling delayed coking processes based on known models from^38,39^ and the system of models of interconnected DCU units developed in this work, the input operating parameters are changed in the allowable range, defined in the process regulations of the DCU Atyrau refinery. For example, the volume of raw materials supplied to the coking reactor ${x}_{1}\in \left[100-108\right],$ t/hour; temperature and pressure in coking reactors, respectively $x_{2} \in \left[ {480 - 490} \right],$ °C; $x_{3} \in \left[ {3.5 - 5.0} \right],$ kg/cm^2^; raw material coking index $x_{4} \in \left[ {8.0 - 10.0} \right]$ and recirculation ratio $x_{5} \in \left[ {1.1 - 1.4} \right]$ and etc.

The main limitations of the proposed approach to computer simulation and optimization of CTS operating modes using the example of DCU include:

-  the complexity of assessing the degree of belonging of fuzzy parameters to fuzzy sets, adequately describing them, and the difficulties in optimizing fast processes, which are due to the lack of time for DM to simulate various operating modes of the object and compare the results to select the best solution.
-   some DM difficulties in the process of choosing a solution.

To eliminate these limitations of the approach, the authors in further research plan to create and use a computer system that supports the process of assessing the degree of belonging of fuzzy indicators to fuzzy sets in an interactive mode with DM. In addition, such a system allows to train and support DM in the process of analysis and selection of the final solution. In addition, to solve the complexity associated with lack of time to simulate different operating modes of the object and compare the results to select the best solution in the future, it can be eliminated by developing and using heuristic decision-making methods for choosing the optimal operating modes of the CTS in a fuzzy environment.

The developed system of DCU models and the computer simulation system can be used in practice for system simulation of DCU operating modes in order to optimize and select the optimal modes of the object of research. To do this, users (operators controlling the DCU operating modes) using the user interface in an interactive mode, changing the values of the input parameters of the object, based on the developed models, simulates various DCU operating modes. At the same time, as a result of modeling using the user interface of the modeling system, the values of the output parameters corresponding to the selected values of the input parameters are displayed in a form convenient for analysis. These output parameters are the criteria that are used to select the DM operating modes of the DCU. The application of the developed DCU models in industry for system modeling and selection of the optimal mode of operation of objects is quite possible. The proposed computer simulation and optimization system based on the developed DCU models has been successfully tested in the DCU Atyrau refinery and accepted for implementation and use in practice.

To apply the developed system of models to other DCUs, it is necessary to identify the parameters of the models according to the data of these DCUs, then the proposed models are quite applicable for modeling and optimizing other DCUs. To apply the proposed models to similar chemical-technological processes, it is necessary, using the proposed method of developing a system of models, first to identify the structures of the models, then their parameters. Thus, in order to apply the developed approach to system modeling to other technological systems, it is necessary to take into account the input parameters that affect the output parameters of the system and are criteria for assessing the quality of the object. In addition, when developing models of objects characterized by a deficit and fuzziness of the initial information, it is necessary to take into account and use the available information of a different nature.

## Conclusion

The main results of the research and conclusions are:

1. Based on a systematic approach, a method is proposed for developing models of interrelated CTS technological units based on available information of a different nature, which makes it possible to create a system of interrelated models;
2. Based on the proposed method for developing a system of CTS models, combined models of the R-1–R-4 coking reactors and the main rectification column C-1, as well as statistical models of the DCU preheating furnaces, were developed;
3. The developed models of the main DCU units for system modeling and optimization of the DCU operating modes are combined into a system of models, taking into account the interconnections of the main units and in accordance with the course of the delayed coking process in the DCU and implemented in software;
4. Computer modeling and optimization of DCU operating modes based on the developed system of models have been carried out. The results of modeling based on the developed system of models, known models and production and experimental data from the DCU Atyrau refinery are compared, the advantages of the proposed approach over known modeling methods are shown.

*The novelty of the results* lies in the development of an effective method for synthesizing a system of models of interrelated CTS units under conditions of uncertainty and fuzziness based on a systematic approach using various types of available information. Due to the use of experience, knowledge and intuition of DM, experts in the development of models and optimization of CTS operating modes and the effect of synergy, the emergence of the resulting system of models, the high efficiency of the proposed approach is ensured.

## Data Availability

Data supporting the results of this study was obtained from the Atyrau Oil Refinery, but restrictions apply to the availability of this data, which was used under license for the current study, and is therefore not publicly available. However, the data is available from the authors upon reasonable request and with the permission of a third party (Atyrau Refinery). To request data from this study in the future, you can contact the author by correspondence: Elmira Dyussembina, e-m-i-k-o_.90@mail.ru.
